# First-line single agent treatment with gefitinib in patients with advanced non-small-cell lung cancer

**DOI:** 10.1186/1756-9966-29-126

**Published:** 2010-09-15

**Authors:** Yong-Mei Yin, Yi-Ting Geng, Yong-Feng Shao, Xiao-Li Hu, Wei Li, Yong-Qian Shu, Zhao-Xia Wang

**Affiliations:** 1 Department of Oncology, The First Affiliated Hospital of Nanjing Medical University, Guangzhou Road. #300, Nanjing 210029, P.R. China; 2 Department of Chest Surgery, The First Affiliated Hospital of Nanjing Medical University, Guangzhou Road. #300, Nanjing 210029, P.R. China; 3 Department of Oncology, The Second Affiliated Hospital of Nanjing Medical University, Jiangjiayuan. #121, Xiaguan District, Nanjing 210011, P.R. China

## Abstract

**Background:**

Lung cancer is a malignant carcinoma which has the highest morbidity and mortality in Chinese population. Gefitinib, a tyrosine kinase (TK) inhibitor of epidermal growth factor receptor (EGFR), displays anti-tumor activity. The present data regarding first-line treatment with single agent gefitinib against non-small-cell lung cancer (NSCLC) in Chinese population are not sufficient.

**Purpose:**

To assess the efficacy and toxicity of gefitinib in Chinese patients with advanced non-small-cell lung cancer (NSCLC), a study of single agent treatment with gefitinib in Chinese patients was conducted.

**Methods:**

45 patients with advanced NSCLC were treated with gefitinib (250 mg daily) until the disease progression or intolerable toxicity.

**Results:**

Among the 45 patients, 15 patients achieved partial response (PR), 17 patients experienced stable disease (SD), and 13 patients developed progression disease (PD). None of the patients achieved complete response (CR). The tumor response rate and disease control rate was 33% and 71.1%, respectively. Symptom remission rate was 72.5%, and median remission time was 8 days. Median overall survival and median progression-free survival was 15.3 months and 6.0 months, respectively. The main induced toxicities by gefitinib were skin rash and diarrhea (53.3% and 33.3%, respectively). The minor induced toxicities included dehydration and pruritus of skin (26.7% and 22.2%, respectively). In addition, hepatic toxicity and oral ulceration occurred in few patients (6.7% and 4.4%2, respectively).

**Conclusions:**

Single agent treatment with gefitinib is effective and well tolerated in Chinese patients with advanced NSCLC.

## Background

Lung cancer is a malignant carcinoma with high morbidity and mortality in Chinese population. Non-small cell lung cancer (NSCLC) accounts for approximately 80% of all lung cancers. The synthetical therapy has been developed remarkably, however the efficacy on locally advanced or metastatic NSCLC is still poor. Recently, the molecular-targeted therapy with gefitinib shows favorable performance. Gefitinib is a tyrosine kinase (TK) inhibitor of epidermal growth factor receptor (EGFR). It blocks signal pathways involved in proliferation and survival of cancer cells [1], and displays activity against malignant tumors. Two large randomised phase II studies (IDEAL1 and 2) in patients with locally advanced or metastatic NSCLC after failure of platinum-based chemotherapy showed a higher response rate of gefitinib (12%-18%) [2,3]. Compared to docetaxel, gefitinib showed superior progression-free survival (PFS), objective response rate (ORR), better tolerability, and similar quality of life (QOL) improvement rates in pretreated NSCLC [4]. Gefitinib was also effective and safe in Chinese patients with recurrent advanced NSCLC [5].

In 2006, Niho et al. reported response rate of 30%, median survival time (MST) of 13.9 months and 1-year survival rate of 55% in advanced NSCLC after first-line single agent treatment with gefitinib[6]. Some other groups also reported that first-line single agent treatment with gefitinib may have better effect in patients with advanced NSCLC than standard first-line chemotherapy [7-10]. Gefitinib showed clinical benefits for EGFR mutation NSCLC patients with extremely poor performance status (PS)[11,12]. The large randomized trial (IPASS research) which compared gefitinib with carboplatin/paclitaxel in patients with advanced NSCLC demonstrated superiority of gefitinib relative to carboplatin/paclitaxel in terms of PFS, ORR, tolerability, and QOL improvement rates. However, the overall survival (OS) and disease-related symptom improvement rates were similar [13]. In 2009, Kim et al. demonstrated that compared to pre-gefitinib eras, the survival of advanced NSCLC patients was significantly improved in post-gefitinib eras in Korea [14].

However, the present data regarding first-line treatment with single agent gefitinib against NSCLC in Chinese population are not sufficient. Here, we conducted a study of single agent treatment with gefitinib in 45 patients with advanced NSCLC in order to assess its efficacy and toxicity in Chinese patients.

## Materials and methods

### Patients

45 patients with histologically or cytologically confirmed stage IIIB or IV NSCLC received gefitinib as first-line treatment between July 2006 and Oct 2008 at the First Affiliated Hospital of Nanjing Medical University. All of these patients were treated initially and had at least one measurable focus according to standard Response Evaluation Criteria in Solid Tumors (RECIST) [15]. These 45 patients consisted of 19 males and 26 females with median age around 61.8 years (range: 30-78). 17 patients had smoking history. In terms of tumor histologic types, the patients included 26 adenocarcinomas, 4 bronchioloalveolar carcinomas, 10 squamous cell carcinomas and 5 adenosquamous carcinomas. According to American Joint Committee on Cancer (AJCC) staging manual, 14 patients were in stage IIIB and 31 patients in stage IV. The Eastern Cooperative Oncology Group Performance Status (ECOG-PS) value was less than 2 in 32 patients, and 3 - 4 in 13 patients (Table 1). All patients provided written informed consent before enrollment. This protocol was approved by the Institutional Review Boards of the participating centers.

**Table 1:** Clinical material and efficacy of the 45 patients

Characters	NO.	CR, n (%)	PR, n (%)	SD, n(%)	PD, n (%)
Gender					
Male	19	0	15.8(3)	36.8(7)	47.4(9)
Female	26	0	46.1(12)	38.5(10)	15.4(4)
Age(year)					
< 70	35	0	34.3(12)	37.1(13)	28.6(10)
≥70	10	0	30.0(3)	40.0(4)	30.0(3)
Smoking status					
Smokers	17	0	17.6(3)	41.2(7)	41.2(7)
Non-smokers	28	0	42.9(12)	35.7(10)	21.4(6)
Tumor histology					
Adeno.	26	0	38.5(10)	42.3(11)	19.2(5)
BAC	4	0	75.0(3)	25.0(1)	0.0(0)
Squamous	10	0	10.0(1)	30.0(3)	60.0(6)
Adenosquamous	5	0	20.0(1)	40.0(2)	40.0(2)
Stage					
IIIb	14	0	28.6(4)	50.0(7)	21.4(3)
IV	31	0	35.4(11)	32.3(10)	32.3(10)
Brain metastasis	4	0	75.0(3)	25.0(1)	0.0(0)
PS value					
≤ 2	32	0	37.5(12)	37.5(12)	25.0(8)
3~4	13	0	23.0(3)	38.5(5)	38.5(5)

### Therapy

Gefitinib (AstraZeneca Company) was administered orally 250 mg daily, 28 days as a cycle. The treatment was continued until disease progression or intolerable toxicity.

### Observation index

We conducted a thorough physical examination on each patient to acquaint with the health status (PS method). Blood routine, hepatic and renal function, electrocardiogram, PET/CT or CT were examined. These indexes were reexamined regularly during the trial, and the image examination was performed after the first one cycle. After that, the image examination was conducted once two cycles. The follow-up of patients by telephone or outpatient service for 1 year was performed.

### Evaluative standards

Tumor response was assessed as complete response (CR), partial response (PR), stable disease (SD), or progression disease (PD) in accordance with the standard of RECIST [15]. A CR was defined as the complete disappearance of all clinically detectable tumors for at least 4 weeks. A PR was defined as an at least 30% decrease in the sum of the longest diameters of the target lesions for more than 4 weeks without new area of malignant disease. PD indicated an at least 20% increase in the sum of the longest diameter of the target lesions or a new malignant lesion. Stable disease was defined as insufficient shrinkage to qualify for PR and insufficient increase to qualify for PD. An objective response rate (ORR) indicated the proportion of patients achieved CR and PR, while a disease control rate (DCR) indicated the proportion of patients achieved CR, PR and SD. Progression-free survival (PFS) was measured from Day 1 of treatment until the first objective or clinical sign of disease progression. Overall survival (OS) was measured from Day 1 of treatment until the date of death. The alteration of patients' symptoms including appetite, fatigue, cough, dyspnea, hemoptysis and pain referencing to Lung Cancer Symptom Scale (LCSS) [16] was observed. Symptomatic remission was considered if the score over 25 points. Symptom remission time means the span from initial administration to symptom remission. Adverse effects including 5 degrees (0-IV) were evaluated following the standard enacted by the World Health Organization in 1981.

### Statistical considerations

The data was analyzed by SPSS11.5. Intergroup comparison was conducted by X2 checking. Survival analyses were performed by Kaplan-Meier method. Survival deviation was calculated by Log-Rank test. All P-values were considered significant if P ≤ 0.05.

## Results

### Clinical efficacy

All of these patients were eligible. None of the patients achieved CR. 15 patients (33.3%) achieved PR and 17 patients (37.8%) had stable disease (SD). 13 patients (28.9%) developed progressive disease (PD). ORR and DCR was 33.3% and 71.1% respectively. Subset analysis according to basic traits of the patients was shown in Table 1. Table 2 showed that the efficacy of gefitinib therapy correlated with gender, tumor histology (P < 0.05). However, other factors such as age, smoking status, disease stage, and ECOG-PS didn't correlate with the efficacy of gefitinib therapy.

**Table 2:** Gradational analysis of ORR and DCR

Characters	ORR(%)	P value	DCR(%)	P value
Gender				
Male	13.3	0.033	52.6	0.019
Female	40.0		84.6	
Age(year)				
< 70	34.3	1.000	71.4	1.000
≥70	30.0		70.0	
Smoking status				
Smokers	17.6	0.082	58.8	0.281
Nonsmokers	42.9		78.6	
Tumor histology				
Adeno. And BAC	43.3	0.044	83.3	0.027
Non-adeno.	13.3		46.7	
Stage				
IIIb	28.6	0.909	78.6	0.699
IV	35.5		67.7	
PS value				
≤ 2	37.5	0.561	75.5	0.589
3~4	23.1		61.5	

It is notable that there were 4 patients with brain metastasis in this trial, including 3 cases of PR and 1 case of SD. Brain metastatic focuses disappeared in 2 patients of PR, and their primary tumor reduced. One of them expressed headache palliative at the day 1. The primary and metastatic tumors of one patient reduced two weeks later.

### Remission of symptoms

In this trial, except 5 patients whose PS = 0, 29 of the other 40 patients (72.5%) achieved palliative symptoms such as fatigue, cough, pain, etc. Remission time arranged from 1 to 14 days, median remission time was 8 days.

### Overall survival

**Figure 1:**
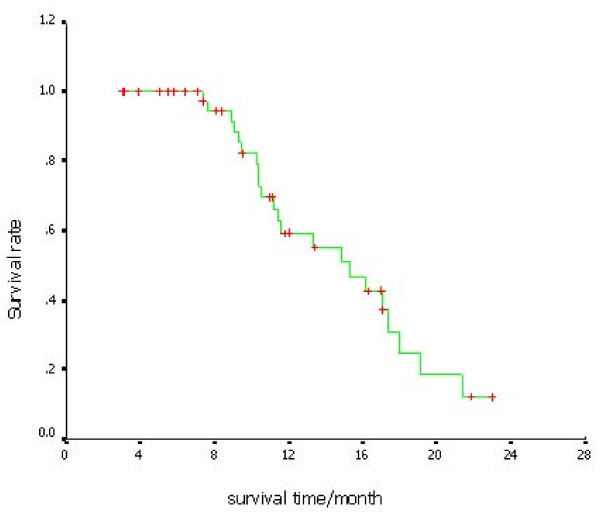
**Kaplan-Meier curve of OS for all patients**. The MST is 15.3 months. 1 year survival rate is 50%.

**Figure 2:**
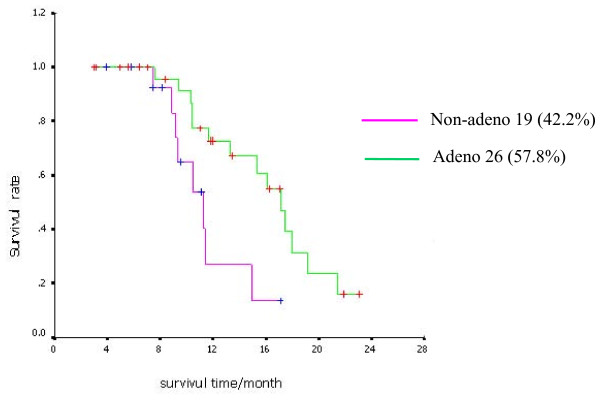
**Kaplan-Meier curve of OS for adenocarcinoma patients (green) and non-adenocarcinoma (pink)**. Adenocarcinoma was remarkably longer than that of non-adenocarcinoma (P = 0.0149).

MST of the 45 patients was 15.3 months by Oct 15, 2008, (95% CI 11.22-19.38). OS arrange from 7.4 to 23 months, and the patient who had the longest OS was still alive at the most recent follow-up. The 1-year survival rate was 50%. The Kaplan-Meier survival curve was showed in Figure 1. The MST of patients with adenocarcinoma and non-adenocarcinoma was 17.1 months (95%CI 14.79-19.41) and 11.2 months (95%CI 8.67-13.73), respectively. The MST of patients with adenocarcinoma was remarkably longer than that of non-adenocarcinoma (P = 0.0149) (Figure 2). Other factors such as gender, smoking status, etc., had no obvious effects on survival (Smokers indicated current or former smokers, and nonsmokers was defined as persons who had never smoked.).

### Progression-free survival time

**Figure 3:**
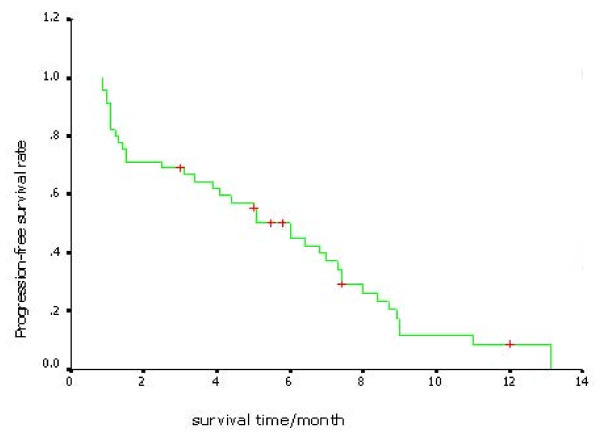
**Kaplan-Meier curve of PFS**. The median PFS was 6.0 months.

The median PFS was 6.0 months, (95% CI 4.36-7.64). Kaplan-Meier curve of PFS was showed in Figure 3.

### Toxicity and adverse effects

**Figure 4:**
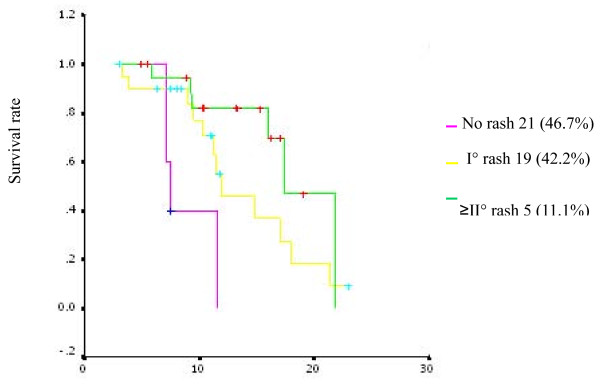
**Kaplan-Meier survival curve of patients with grade 0 to 3 acne-like rash**.

As shown in Table 3, the most common toxicities of gefitinib treatment were rash (53.3%) and diarrhea (33%). In addition, 26.7% and 22.2% of the patients showed dehydration and pruritus of skin. 6.7% of the patients showed Grade 2 or 3 hepatic toxicity. 4.4% of the patients (2 persons) showed oral ulcer. No patients developed interstitial lung disease (ILD). Most of the toxicity was grade 1 to 2, and remitted after treatment. Grade 3 rash of one patient was remitted by reducing the dose of gefitinib. The relationship between rash and OS is showed in Figure 4.

**Table 3:** Assessment of toxicity (case, %)

Toxicity	Grade(WHO)				
	0	I	II	III	IV
Rash	21(46.7)	19(42.2)	4(8.9)	1(2.2)	0(0)
Pruritus	35(77.8)	10(22.2)	0	0	0
Dry skin	33(73.3)	11(24.4)	1(2.2)	0	0
Diarrhea	30(66.7)	13(28.9)	2(4.4)	0	0
Oral ulcer	43(95.6)	2(4.4)	0	0	0
Nausea/vomit	37(82.2)	8(17.8)	0	0	0
Hepatic toxicity	42(93.3)	1(2.2)	2(4.4)	0	0
Interstitial lung Disease(ILD)	45(100.0)	0	0	0	0

## Discussion

Because of high morbidity and mortality, investigators pay more attentions to the therapy of lung cancer in recent years. Platinum-based combination chemotherapy has been the standard first-line therapy for advanced NSCLC. However, it brings about severe adverse effects such as vomiting, renal toxicity, cytopenia, etc.. Recently, molecular-targeted agents have been introduced in the treatment of NSCLC. Gefitinib, a tyrosine kinase inhibitor of EGFR, has been allowed to treat NSCLC clinically. The second-line treatment with gefitinib has response rate, survival benefit and safety not inferior to chemotherapy. Two trials in patients who previously failed platinum-based chemotherapy, IDEAL-1 and 2, revealed a favorable ORR (12-18%), a DCR of 50%, and good tolerability of gefitinib treatment [2,3]. Gefitinib have been suggested to have better efficacy in patients of females or non-smokers, patients with adenocarcinoma (particularly with bronchioloalveolar carcinoma), patients with previous immune/endocrine therapy, and patients with a PS of 0 or 1[2]. A trial about the treatment of NSCLC patients from Asia with gefitinib resulted in an ORR more than 25% and a DCR more than 60% [17]. Recently, Lee et al. [5] demonstrated that, as second-line therapy, gefitinib has superior PFS, better tolerability, and similar QOL improvement rates compared to docetaxel.

Nowadays, more and more clinical investigations have been carried out to evaluate the efficacy of gefitinib as first-line treatment of advanced NSCLC. Niho et al.[6] reported a response rate of 27% with gefitinib treatment in 40 patients with advanced NSCLC. Yang et al.[18] from Taiwan reported that first-line treatment with gefitinib in 196 patients with NSCLC achieved an ORR of 42%, a DCR of 61%, and a 1-year survival rate of 47.5%. A large phase III trial IPASS, which was designed to compare gefitinib as first-line treatment of NSCLC patients with standard chemotherapy, demonstrated superiority of gefitinib in terms of 12-month rates of PFS (24.9% vs. 6.7%, P < 0.05), ORR (43.0% vs. 32.2%, P = 0.0001), and tolerability profile compared with carboplatin plus paclitaxel. Recently, Maemondo et al.[9] reported that the gefitinib group had a significantly longer median PFS (10.8 months vs. 5.4 months; P < 0.001), as well as a higher response rate (73.7% vs. 30.7%, P < 0.001) than the standard chemotherapy group. A study conducted in Japan also showed a longer PFS in gefitinib group than the cisplatin plus docetaxel group (9.2 months vs. 6.3 months, P < 0.0001) [10]. In our study of first-line treatment with gefitinib in Chinese patients with advanced NSCLC, we obtained an ORR of 33.3%, a DCR of 71.1%, a median PFS of 6.0 months, and a median OS of 15.3 months. These results were compatible with the reports aforementioned.

The IPASS study suggested that gefitinib would be efficacious in first-line treatment of locally advanced or metastatic NSCLC patients with adenocarcinoma who have never or seldom smoked [13]. Consistent with this result, we found that females and patients with adenocarcinoma (including bronchioloalveolar caicinoma) were more sensitive to gefitinib. Although the response rate of gefitinib in non-smokers seemed higher than that in smokers, the result had no statistical significance due to the small sample size. The OS of patients with adenocarcinoma was longer than that of patients with non-adenocarcinoma (17.1 months vs. 11.2 months, P = 0.0149). However, other factors such as gender and smoking status have no obvious correlation to OS. In addition, we found that the OS of patients with rash was longer than that of patients without rash, and a longer OS was coupled with greater rash. Because there were few cases with grade 2 or more serious rash, this result needs to be verified further. Moreover, our study showed favorable efficacy of gefitinib in patients with brain metastasis.

Gefitinib is well tolerated in advanced NSCLC. The common adverse effects of gefitinib were skin rash, diarrhea, anorexia, elevated aminotransferase lever, and interstitial lung disease, etc [9-11,19]. Similarly, mild toxicities including skin rash (53.3%), diarrhea (33%), Grade 2 or 3 hepatic toxicity (6.7%), and oral ulcer (4.4%) were observed in our study. No patients developed ILD. Since the tolerance of gefitinib in NSCLC is better than chemotherapy, and gefitinib could provide clinical benefits for patients with extremely poor PS [11,12], it may be a better choice to treat patients who can't tolerate chemotherapy compared to best supportive care (BSC).

It has been recently reported that the sensitivity and survival benefit of gefitinib treatment was higher in NSCLC patients with EGFR mutations than the patients without EGFR mutations [20-22]. Chinese patients of lung cancer have a higher frequency of EGFR mutations than American patients. As a result, Chinese patients were much more sensitive to gefitinib than Americans [23]. Besides mutations, gene copy number and polymorphism of EGFR were also related to the responsiveness of gefitinib in advanced NSCLC [24,25]. EGFR mutations of NSCLC patients can be detected using plasma and pleural effusion samples, which provides a noinvasive method to predict the efficacy of gefitinib in advanced NSCLC [26]. Detecting the mutations of EGFR plays an important role in guiding the first-line treatment with gefitinib in patients with advanced NSCLC. Besides EGFR mutations, the favorable PFS after gefitinib treatment was also associated with high levels of serum surfactant protein D (SP-D) [27]. In future studies, we will investigate the molecules which affect and (or) can be used to predict the efficacy of gefitinib in NSCLC.

## Conclusions

Single agent treatment with gefitinib is effective in patients with advanced NSCLC, and well tolerated in Chinese patients. Gefitinib could be used as first-line treatment for specific subgroups of NSCLC such as females, non-smokers, and patients with adenocarcinoma.

## Abbreviations

NSCLC: non-small-cell lung cancer; TK: tyrosine kinase; EGFR: epidermal growth factor receptor; CR: complete response; PR: partial response; SD: stable disease; PD: progression disease; PFS: progression-free survival; ORR: objective response rate; QOL: quality of life; PS: performance status; ECOG-PS: Eastern Cooperative Oncology Group performance status; DCR: disease control rate; OS: overall survival; RECIST: Response Evaluation Criteria In Solid Tumors; LCSS: Lung Cancer Symptom Scale; MST: median survival time; ILD: interstitial lung disease; BSC: best supportive care; TTP: time to progression; SP-D: serum surfactant protein D

## Competing interests

The authors declare that they have no competing interests.

## Authors' contributions

YQS contributed to conception and design, and gave final approval of the version to be published. ZXW contributed to conception and design. YMY acquired the data and revised the manuscript critically for important intellectual content. YTG acquired the data and drafted the manuscript. YFS acquired the data. XLH and WL contributed to statistic analysis. All authors have read and approved the final manuscript.
